# Whole-Brain Three-Dimensional Profiling Reveals Brain Region Specific Axon Vulnerability in 5xFAD Mouse Model

**DOI:** 10.3389/fnana.2020.608177

**Published:** 2020-11-26

**Authors:** Jianping Zhang, Ben Long, Anan Li, Qingtao Sun, Jiaojiao Tian, Ting Luo, Zhangheng Ding, Hui Gong, Xiangning Li

**Affiliations:** ^1^Britton Chance Center for Biomedical Photonics, Wuhan National Laboratory for Optoelectronics, MoE Key Laboratory for Biomedical Photonics, School of Engineering Sciences, Huazhong University of Science and Technology, Wuhan, China; ^2^HUST-Suzhou Institute for Brainsmatics, JITRI Institute for Brainsmatics, Suzhou, China; ^3^CAS Center for Excellence in Brain Science and Intelligence Technology, Chinese Academy of Sciences, Shanghai, China

**Keywords:** Alzheimer’s disease, early stage, axonopathy, whole-brain imaging, three-dimension, medial mammillary nucleus

## Abstract

Axonopathy is a pathological feature observed in both Alzheimer’s disease (AD) patients and animal models. However, identifying the temporal and regional progression of axonopathy during AD development remains elusive. Using the fluorescence micro-optical sectioning tomography system, we acquired whole-brain datasets in the early stage of 5xFAD/Thy1-GFP-M mice. We reported that among GFP labeled axons, GFP-positive axonopathy first formed in the lateral septal nucleus, subiculum, and medial mammillary nucleus. The axonopathy further increased in most brain regions during aging. However, most of the axonopathic varicosities disappeared significantly in the medial mammillary nucleus after 8 weeks old. Continuous three-dimensional datasets showed that axonopathy in the medial mammillary nucleus was mainly located on axons from hippocampal GFP-positive neurons. Using the rabies viral tracer in combination with immunohistochemistry, we found that axons in the medial mammillary nucleus from the subiculum were susceptible to lesions that prior to the occurrence of behavioral disorders. In conclusion, we created an early-stage spatiotemporal map of axonopathy in 5xFAD/Thy1-GFP-M mice and identified specific neural circuits which are vulnerable to axon lesions in an AD mouse model. These findings underline the importance of early interventions for AD, and may contribute to the understanding of its progression and its early symptom treatment.

## Introduction

Alzheimer’s disease (AD) is a neurodegenerative disease characterized by progressive memory loss and cognitive impairment (Canter et al., 2016), which are both associated with synaptic loss and neuronal degeneration (Long and Holtzman, 2019). Axonopathy has been described as one of the pathological features of AD, observed in both AD patients and animal models (Stokin et al., 2005; De Vos et al., 2008). In vivo studies showed that axons from neocortical and hippocampal projection neurons were damaged in APP-PS1/Thy1-GFP-M mice (Blazquez-Llorca et al., 2017) and APP-PS1/Thy1-YFP mice (Tsai et al., 2004). These impaired axons developed large varicosities with diameters larger than 3.0 μm, and the condition was named axonopathy (Stokin et al., 2005; Weber et al., 2019). Immunohistochemical staining and ultrastructural analysis revealed aberrant accumulation of axonal cargo within the locally swollen axons, including microtubule-binding proteins, neurotransmitters, mitochondria, and other organelles (Gowrishankar et al., 2015; Sadleir et al., 2016). These pathological changes were reported to occur at different time points in AD animal models. In TgCRND8/Thy1-YFP mice, for instance, cortical projection neurons showed axonopathy at 2 months old (Adalbert et al., 2009), while, it happened in 4-months-old PSAPP/Thy1-YFP mice (Tsai et al., 2004). These differences may be related to the different strains of experimental animals used in these studies. Furthermore, in these studies, the temporal and spatial progression of the axonopathy was not reported. It remains unclear whether all neural circuits are equally vulnerable to axon lesions or specific neural circuits are more vulnerable. Therefore, confirming the temporal and spatial progression of axonopathy at the whole-brain level is important for understanding the pathological development of impaired brain functions and for determining early treatment strategies in AD mouse models, which may in turn shed light on the possible treatments to alleviate the symptoms of AD patients at an early stage.

Transgenic animal models for AD have promoted research on the pathogenesis of AD. Compared to other AD transgenic mice, the 5xFAD mouse model rapidly develops severe amyloid pathology, neuron loss, and cognitive memory impairment (Oakley et al., 2006). This model has been widely used to study the pathogenesis of AD, including researching the effects of amyloid plaques on the vulnerability of subcortical brain regions (Canter et al., 2019), analyzing the damage to cholinergic neurons (Yan et al., 2018), revealing the relationship between Amyloid-β and Tau protein (He et al., 2018), and the mechanisms underlying axonal damage (Sun et al., 2014; Sadleir et al., 2016). In addition, axonopathy of projection neurons in the neocortex and hippocampal formation was found in 4–6 months old of 5xFAD mice (Buskila et al., 2013; Sadleir et al., 2016). Hence, this model can show axonopathy and rapidly develop major AD-like pathologic phenotype. On the other hand, the Thy1-GFP-M mice under the neural-specific *Thy1* promoter were widely used in vivo and in vitro studies (Gong et al., 2016; Blazquez-Llorca et al., 2017). Previous studies have confirmed that Thy1-GFP-M mice can specifically label neurons with sparse, stable and strong fluorescent labeling (Feng et al., 2000; Guo et al., 2017). The continuous GFP-labeled axons can be traced throughout regions (Gong et al., 2016) and individual axonopathic swelling can be identified without interference from other fluorescent signals by crossing Thy1-GFP-M and AD mice (Blazquez-Llorca et al., 2017). Therefore, in the present study, we crossed the 5xFAD with Thy1-GFP-M mice (5xFAD/GFP mice) for axonopathy mapping.

Previous studies on axonopathy were based on two-dimensional images, and mainly focused on a small number of brain areas, such as the hippocampal formation and neocortex (Tsai et al., 2004; Adalbert et al., 2009). Therefore, more comprehensive analysis is needed on the spatiotemporal variations of axonopathy to reveal unexplored axonal lesions and determine which brain areas are more vulnerable to axonopathy. Three-dimensional imaging techniques may reveal new details about the structures of complex neural systems. The rapid development of optical imaging systems, such as fluorescence micro-optical sectioning tomography (fMOST) (Gong et al., 2016), serial two-photon tomography (Economo et al., 2016) and FAST (Seiriki et al., 2017), have indeed allowed us to analyze the fine-scale structure of neurons at the whole-brain three-dimensional level.

Using the latest fMOST developed in our laboratory, we performed whole-brain imaging at subcellular resolution (0.32 × 0.32 × 2 μm^3^) to obtain continuous three-dimensional datasets of the distribution of GFP labeled axonopathy at 6-, 7-, 8-, and 16-week-old 5xFAD/GFP mice, respectively. Furthermore, through rabies virus (RV) tracing, we found that the axons projecting to the medial mammillary nucleus (MM) from the subiculum (SUB) were particularly susceptible to lesions that prior to the occurrence of behavioral disorders. Hence, this work provided a spatiotemporal map of axonopathy, and highlighted axonal vulnerability in the SUB-MM circuit during the early stage of the AD model. This might facilitate the understanding of the development of the disease and offer novel treatment options for early AD symptoms.

## Materials and Methods

### Animals and Sample Preparation

The 5xFAD mouse line expresses the human APP and PSEN1 transgenes, with a total of five FAD mutations: *APP KM670/671NL (Swedish), APP I716V (Florida), APP V717I (London), PSEN1 M146L, and PSEN1 L286V* (Oakley et al., 2006). The Thy1-GFP-M mouse line has been previously described (Feng et al., 2000). Heterozygous male 5xFAD mice were crossed with female Thy1-GFP-M mice. The 6-, 7-, 8-, and 16-week-old female 5xFAD mice, and the corresponding Thy1-GFP-M mice and C57 mice control groups, were used for our experiments. All animal experiments complied with the guidelines of the Animal Care and Use Committee of Huazhong University of Science and Technology.

### Amyloid-β Plaque Staining

For the whole-brain staining of amyloid-β plaques (6W, *n* = 3; 7W, *n* = 6; 8W, *n* = 6; 16W, *n* = 6), each brain was stained via immersion in a graded DANIR-8c (Beijing Shihong Pharmaceutical Center, Cat. 072701) staining solution A for 1 days, B for 3 days and C for 1 day (Long et al., 2019). After that, the brains were then rinsed in a 70% alcohol solution for 6 h, embedded, and finally subjected to whole-brain imaging. The solution A: 15% sucrose (w/v) + 0.01 mol/ml DANIR-8c +0.1%Triton X-100+0.05% sodium azide; the solution B: 30% sucrose (w/v) + 0.01 mol/ml DANIR-8c +0.1%Triton X-100+0.05% sodium azide; the solution C: 0% sucrose (w/v) + 0.01 mol/ml DANIR-8c +0.1%Triton X-100+0.05% sodium azide.

### Whole-Brain Imaging and Image Processing

As described in previous works (Gong et al., 2016; Li et al., 2018), each sample was embedded in resin. After that, the sample was automatically imaged at a voxel resolution of 0.32 × 0.32 × 2 μm^3^, to acquire whole-brain datasets through fMOST (40x, 0.8NA, Wuhan OE-Bio Co., Ltd., China). The raw images were pre-processed on a computing server (72 cores, 2 GHz/core) and a graphical workstation (Dell, Round Rock, TX, United States; T7920). Briefly, the adjacent overlap of different raw images was removed and stitched to an entire section, and calibration for uneven illumination was performed through a projection curve, using our customized program.

### Quantitative Analysis and Reconstruction of Axonopathy

For the quantitative analysis in multi brain regions (*n* = 3 for 8W and 16W, respectively), the diameter of axonopathic swellings were defined as larger than 3 μm (Stokin et al., 2005; Weber et al., 2019). The pre-processed images were performed a projection of every 20 μm and were manually counted using the Cell Counter ImageJ plug-in (Version 1.48, NIH, United States).

The three-dimensional datasets of the regions of interest were imported into the Amira software (Version 6.1.1, FEI, France), at a voxel resolution of 0.32 × 0.32 × 2 μm^3^, for reconstruction, and saved as tiff files. Then, axonopathy (6W, *n* = 6; 7W, *n* = 8; 8W, *n* = 8; 16W, *n* = 8) and amyloid-β plaque morphology were reconstructed and counted automatically via the surface creation feature of the Imaris software (Version 9.0, Bitplane, Switzerland). The results were manually checked to exclude the mislabeled cell bodies by the software. The axonopathy with a size ≤ 100 μm^2^ and >100 μm^2^ were defined as small and large, respectively. The amyloid-β plaques with a size ≤ 100 μm^2^, between 100 μm^2^ and 500 μm^2^, and ≥500 μm^2^ were defined as small, medium and large, respectively.

### Stereotaxic Injections

All mice were deeply anesthetized by intraperitoneal injection (100 g/ml) of 2% chloral hydrate and 10% urethane-configured anesthetic, and then mounted and micro-injected with a stereotaxic system. RV-DG-N2C-DsRed (Zhu et al., 2019) was purchased from Brain VTA. RV-DG-N2C-DsRed (150 nl, 5 × 107 IFU/mL) was injected into the MM area (bregma, -2.83 mm; lateral, 0.0 mm; depth, -5.17 mm from the skull surface) both at 15-week-old 5xFAD and C57 mice (*n* = 4 for each group). All viral injections were performed through a glass micropipette, at a speed of 20 nl/min, using a microsyringe pump. After completion of each viral injection, the needle was held in place for 10 min, and then slowly retreated. After that, the incisions were stitched, and lincomycin hydrochloride and lidocaine hydrochloride gel were applied to the animals, to reduce inflammation and pain. The mice injected with RV underwent further experiments seven days after the viral injections.

### Immunohistochemistry and Cell Counting

The mice were anesthetized by intraperitoneal injection (100 g/ml) of 2% chloral hydrate and 10% urethane-configured anesthetic, and then perfused with 0.01 M PBS (Sigma-Aldrich Inc., St. Louis, United States) for 10 min, followed by perfusion with 4% PFA for 10 min. The brains were removed and post-fixed in 4% PFA solution at 4°C for 24 h. For immunohistochemistry, the brains were cut in 70-μm-thick coronal sections using a vibratome (Leica, VT1200). The brain sections were rinsed in 0.01 M PBS (3 × 10 min), and blocked with 5% (wt/vol) bovine serum albumin in 0.01 M PBS (at 37°C for 2 h). Next, the brain sections were incubated with a primary antibody (at 4°C for 48 h): anti-NeuN (1:800, Rabbit, Abcam, ab7349). After this step, the sections were rinsed with 0.01 M PBS (3 × 10 min), then incubated with a fluorophore-conjugated secondary antibody (1:800, at 37°C for 2 h): Alexa Fluoro-488, Goat anti-Rabbit IgG. Following this procedure, the brain sections were rinsed in 0.01 M PBS (3 × 10 min), and were finally mounted on glass slides, to be imaged with a commercial confocal microscope (Carl Zeiss, LSM710). The RV-labeled neurons and NeuN-positive neurons were manually counted using the Cell Counter ImageJ plug-in (Version 1.48, NIH, United States).

### Behavioral Assays

The 8-week-old and 16-week-old female mice were conducted to behavioral experiments between 8:00 AM and 6:00 PM. All the experimental mice were transferred to the behavior testing room 3 days prior to beginning the first trial to habituate to the condition of the behavior testing room. Wiping the chamber with a 75% ethanol prior to use and before subsequent tests to remove any scent clues left by the previous subject mice.

Open field test (OFT). Mice were individually placed facing one of the walls of a Plexiglass open-field (40 × 40 × 30 cm). Mice was allowed to explore freely for 15 min and the time spent in the outer zone was automatically tracked using EthoVision XT 12.0 (Noldus Apparatus) (8W, *n* = 14 for 5xFAD, *n* = 11 for WT; 16W, *n* = 31 for 5xFAD, *n* = 28 for WT).

Novel-object recognition test (ORT) and object-location recognition (OLT). The test includes two sessions of one trial each: acquisition and retrieval trials. For three consecutive days, the mice was individually habituated to the open field for 10 min. For the ORT (8W, *n* = 14 for 5xFAD, *n* = 13 for WT; 16W, *n* = 27 for 5xFAD, *n* = 20 for WT), mice was placed in an arena that contained two identical objects for 10 min in the acquisition trial. The retrieval session was done 10 min after the acquisition trial. In this trial, one of the objects presented in the first trial was placed with a novel object. Then placed mice back in the arena to explore freely for 10 min. For the OLT (8 W, *n* = 10 for 5xFAD, *n* = 11 for WT; 16 W, *n* = 9 for 5xFAD, *n* = 18 for WT), mice was placed in an arena that contained two identical objects for 10 min in the acquisition trial. The retrieval session was done 10 min after the acquisition trial. In this trial, one of the objects presented in the first trial was removed to a novel location (displaced). Then placed mice back in the arena to explore freely for 10 min. Time spent in active exploration of the each object during the retrieval trial was calculated using EthoVision XT 12.0 (Noldus Apparatus). The mice that did not explore the objects for 20 s within the 10 min period were excluded from the further experiments. A mice was scored as approaching with the object when its nose within 2 cm of the object. Recognition memory was socred using a recognition index for each mouse with a formular (Tnovel or Tfamiliar)/(Tnovel + Tfamiliar) x 100% or (Tdisplaced or Tunmoved)/(Tdisplaced + Tunmoved) x 100%. Delta recognition index was defiened as follows: (Tnovel - Tfamiliar)/(Tnovel + Tfamiliar) x 100% or (Tdisplaced - Tunmoved)/(Tdisplaced + Tunmoved) x 100%.

### Statistical Analysis

Statistical significance was analyzed using GraphPad Prism version 8.0 (GraphPad, La Jolla, CA, United States). All data were presented as mean ± SEM. Statistical comparisons were determined using the Student’s *t*-test and ANOVA.

## Results

### Axons Show Pathology During Early Stage of 5xFAD/GFP Mice

**FIGURE 1:**
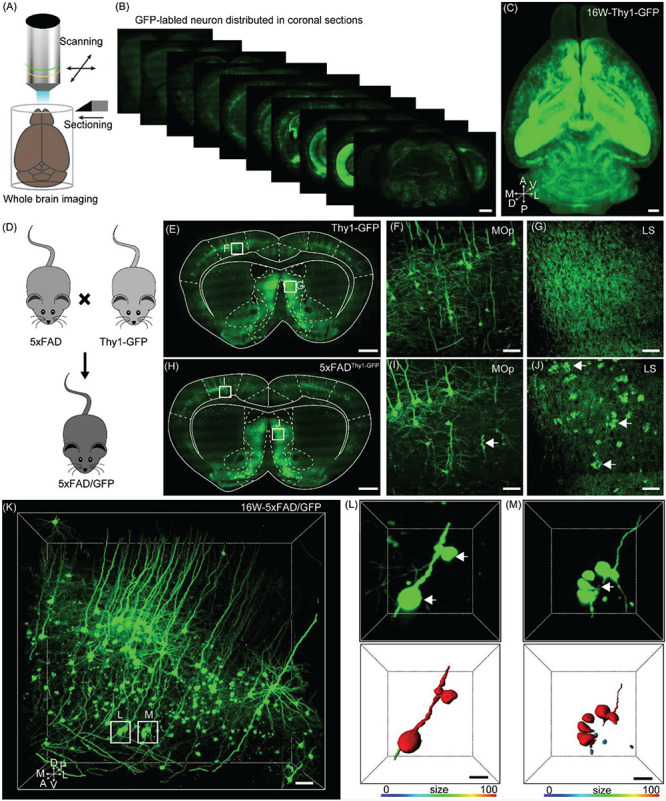
Evaluation axonopathy during early stage of 5xFAD/GFP mice. **(A)** Diagram of the whole-brain imaging procedure. **(B)** Continuous coronal sections taken at 800 μm intervals from a Thy1-GFP-M mice. The thickness of the projection was 20 μm. Scale bar: 1 mm. **(C)** 3D-reconstructed image of GFP-positive neurons in the whole brain of a Thy1-GFP-M mice. A, anterior; D, dorsal; L, lateral; M, medial; P, posterior; V, ventral. Scale bar: 1 mm. **(D)** Schematic diagram illustrating that the neurons were labeled by crossing the 5xFAD mice with the Thy1-GFP-M mice. **(E)** A coronal brain section revealed that there was no axonopathy from 16-week-old Thy1-GFP-M mice. The thickness of the projection was 20 μm. Scale bar: 1 mm. **(F,G)** The magnifications of the primary motor area and LS in E (white squares), respectively. Scale bar: 50 μm. **(H)** A coronal brain section form 16-week-old 5xFAD/GFP mice. The thickness of the projection was 20 μm. Scale bar: 1 mm. **(I,J)** The magnifications of the primary motor area and LS in H (white squares), respectively. The GFP-positive axonopathic swellings were highlighted with white arrows Scale bar: 50 μm. **(K)** 3D image of GFP-positive neurons and fibers in the somatosensory areas from 16-week-old 5xFAD/GFP mice. The axonopathic swellings were highlighted with white boxes. Scale bar: 100 μm. **(L,M)** The three-dimensional image of axons (top) and the surface render of axons showed three-dimensional morphology of axons (bottom) in a 5xFAD/GFP mice. The axonopathy was highlighted with white arrow. Heat-map plots showed the size of axonopathy. Red = bigger size, blue = smaller size. Scale bars: 10 μm both in **(H,I)**.

To locate the neuronal soma and identify the individual axon throughout the entire brain without losing crucial information, we generated whole-brain three-dimensional datasets of GFP-positive neurons in 16-week-old Thy1-GFP-M mice by fMOST at a voxel resolution of 0.32 × 0.32 × 2 μm^3^ (Figures 1A,B). Through these continuous three-dimensional datasets, we found that the vast majority of GFP-positive neurons were located in the deep layer of the cortex, hippocampus, and amygdala (Figure 1C), which is consistent with previous studies (Feng et al., 2000; Porrero et al., 2010).

To evaluate axonal pathological changes in AD, we crossed 5xFAD with Thy1-GFP-M mice (Figure 1D). Thus, we could analyze axonal morphological changes occurring during aging in the mouse model by using the fMOST system. Previous studies have reported that the presence of abnormal axonal morphology in the primary motor cortex neurons of 4–6 month old of 5xFAD mice (Buskila et al., 2013; Sadleir et al., 2016). To determine where and how these detrimental changes appear, we performed whole-brain imaging in 16-week-old 5xFAD/GFP mice. Based on the whole-brain three-dimensional datasets acquired via the fMOST system, we compared the axonal morphological differences between 5xFAD/GFP mice and Thy1-GFP-M mice. At the same age, compared to the Thy1-GFP-M mice (Figures 1E–G), the 5xFAD/GFP mice exhibited numerous axons with swellings or spheroids in cortical and subcortical regions (Figure 1H), such as the primary motor area (Figure 1I) and the lateral septal nucleus (LS) (Figure 1J). In previous reports, this was observed at later time points (Buskila et al., 2013; Sadleir et al., 2016). To further characterize these pathological changes, we reconstructed the spheroids on the axons of GFP-positive neurons in the somatosensory cortex (Figures 1K–M). The individual axon had multiple pathological sites (Figure 1L). Discontinuity of the axon was visible around the clustered swellings (Figure 1M), suggesting axonal disruption as reported in previous studies (Tsai et al., 2004; Adalbert et al., 2009). Our results indicated that axons were severely damaged in 16-week-old 5xFAD/GFP mice.

### Spatiotemporal Axonopathy Patterns During Early Stage of 5xFAD/GFP Mice

**FIGURE 2:**
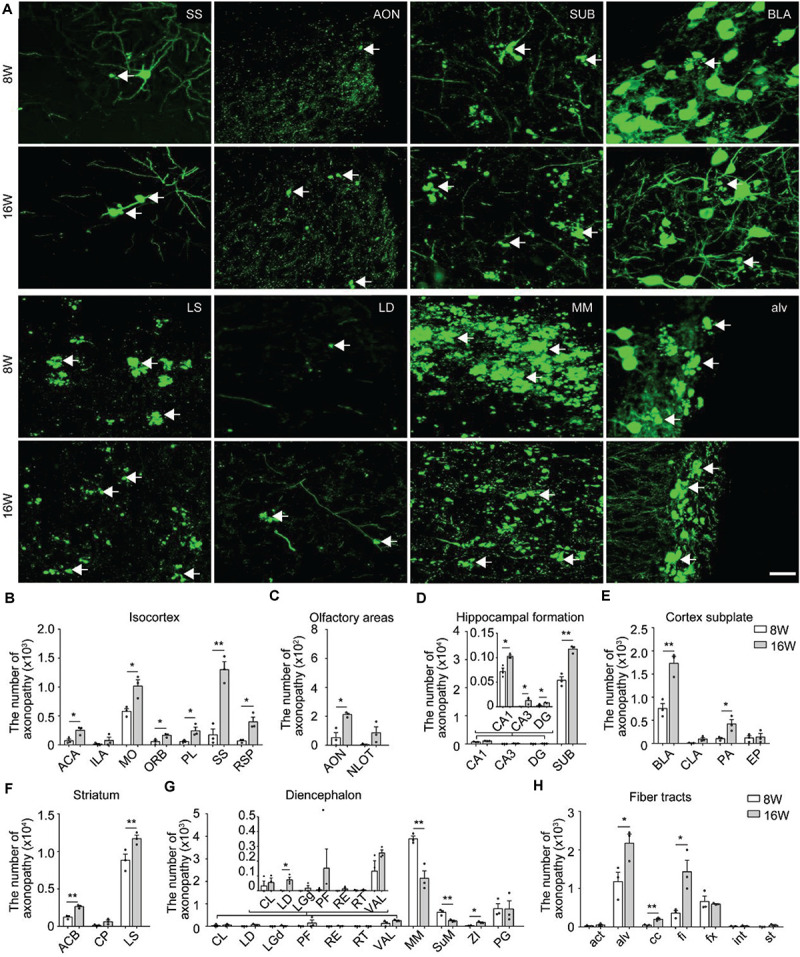
Distribution and comparison of axonopathy in 5xFAD/GFP mice. **(A)** Maximum intensity projection of the axonopathy in representative brain regions from 8- and 16-week-old 5xFAD/GFP mice. Representative axonopathic swellings were highlighted with white arrows. The thickness of the projection was 20 μm. Scale bars: 20 μm. **(B–H)** Quantitative analysis of axonopathy in different subregions between 8- and 16-week-old 5xFAD/GFP mice, respectively (*n* = 3 for each group). All error bars represented mean ± SEM and the significant differences were indicated by *p* value (Student’s *t*-test, *P < 0.05; **P < 0.01).

To study the regional burden and temporal progression of axonopathy more comprehensively at a high spatial resolution, we performed whole-brain imaging from 8-week-old and 16-week-old 5xFAD/GFP mice. As expected, the axonopathy appeared in specific regions during 8 to 16 weeks old, including the isocortex, olfactory area, hippocampal formation, cortical subplate, striatum, thalamus, hypothalamus and fiber tracts (Figure 2A and Supplementary Figure S1). Interestingly, we found that the number of axonal swellings in many brain regions increased, while it seemed to decrease significantly in the MM from 8 to 16 weeks old (Figure 2A).

To better understand the temporal progression of axonal swellings in specific brain regions, we quantified the number of axonopathy in different brain regions in both 8-week-old and 16-week-old 5xFAD/GFP mice.

In the isocortex, region-specific quantification showed that, at 8 weeks old, the somatomotor area contained more axonopathy than other cortical areas (Figure 2B). During 8–16 weeks old, the axonopathy in most cortical areas significantly increased (Figure 2B). In the olfactory areas, axonopathy mainly appeared in the anterior olfactory nucleus and the nucleus of the lateral olfactory tract. The number of axons exhibiting axonopathy in both areas increased during aging (Figure 2C). In the hippocampal formation, axonopathy appeared in the CA1, CA3, dentate gyrus and SUB at 8 weeks old and the number of axons showing axonopathy further increased at 16 weeks old (Figure 2D). Notably, the SUB harbored the vast majority of axons exhibiting axonopathy which was ten times more numerous than in other hippocampal regions since 8 weeks old (Figure 2D), indicating that axons in this area suffered more severe damage. Such axonal damage in the SUB may severely impair information exchange between the hippocampal formation and other brain regions (Roy et al., 2017; Matsumoto et al., 2018).

In the cortex subplate, axonopathy mainly appeared in the basolateral amygdala nucleus. A small number of axonopathy-exhibiting axons appeared in the claustrum, posterior amygdala nucleus and endopiriform nucleus (Figure 2E). During aging, the number of axons exhibiting axonopathy in the basolateral and posterior amygdala nucleus further increased but not in the claustrum and endopiriform nucleus (Figure 2E). In the striatum, the LS contained the most of the axonopathy, and the number of such swellings continued to increase from 8 to 16 weeks old in the caudoputamen and LS (Figure 2F).

In the diencephalon, the mammillary nucleus including the MM and supramammillary nucleus contained the most of axonopathy-exhibiting axons while only a small number appeared in the thalamus (Figure 2G). In contrast to most brain regions, the amount of axonopathy in the MM and supramammillary nucleus significantly decreased from 8 to 16 weeks old (Figure 2G). In particular, for the MM, there was an almost 50% decrease in axonopathy (Figure 2G).

At 8 weeks old, a small number of axonopathy was found in different fiber tracts, such as the alveus, corpus callosum, fimbria, and columns of the fornix, which further increased during aging (Figure 2H). By 16 weeks old, axonopathy in the alveus, corpus callosum and fimbria was more prominent than that at 8 weeks old, respectively (Figure 2H). These data indicated serious fiber tract damage, which may disrupt neural circuits since primary function of these fiber tracts are to connect different areas of the brain (Rajasethupathy et al., 2016).

Overall, the spatiotemporal map of axonopathy supports a model where the subcortical structures – the LS, MM and SUB were most prone to axonopathy in our AD mouse model (Figures 2D,F,G), indicating that axonopathy did not occur uniformly along the axons in different brain areas.

### Three-Dimensional Profiling of Axon Vulnerability in the LS, SUB, and MM

**FIGURE 3:**
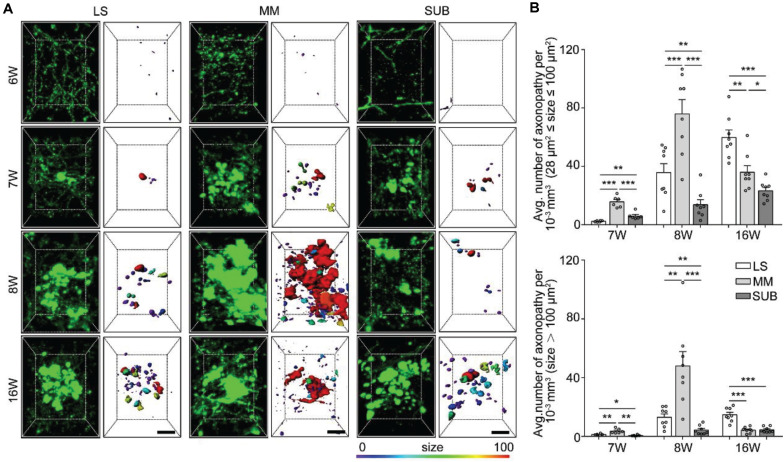
Quantitative analysis of spatiotemporal characteristics of axonopathy in the LS, MM, and SUB during the early stage of 5xFAD/GFP mice. **(A)** A 3D image of axons in the LS, MM and SUB of 5xFAD/GFP mice (left) at 6, 7, 8, and 16 weeks old, respectively. The surface render of axonopathy showed 3D morphology of axonopathic varicosities (right). Heat-map plots showed the size of varicosities. Red = bigger size, blue = smaller size. Scale bars: 10 μm. **(B)** Density comparison of axonopathy among the LS, MM, and SUB at 6, 7, 8, and 16 weeks old, respectively (6W, *n* = 6; 7W, *n* = 8; 8W, *n* = 8; 16W, *n* = 8). All error bars represented mean ± SEM and the significant differences were indicated by *p* value (Student’s *t*-test, *P < 0.05; **P < 0.01; ***P < 0.001).

The above results showed that axonopathy was highest in the LS, SUB, and MM during the early stage of 5xFAD/GFP mice (Figures 2D,F,G), indicating that axons in these brain areas suffered the earliest and most serious damage during the early stage of 5xFAD mice. The three brain regions are cognition-related areas and make dense connections with the hippocampus and cortex (Vann and Aggleton, 2004; Calhoon and Tye, 2015; Matsumoto et al., 2018). Thus, we wanted to determine the spatial distribution and temporal progression of axonopathic swellings in the three brain regions, which would help better understand the spatiotemporal patterns of axonopathy development. We applied fMOST to acquire datasets of GFP-positive neurons back to the earliest time points, from 6 to 7-week-old 5xFAD/GFP mice. Next, we performed three-dimensional reconstruction of axonopathy in the LS, MM and SUB at 6, 7, 8, and 16 weeks old, respectively (Figure 3A). No axonopathy was observed in the entire brain at 6 weeks old (Figure 3A). However, it only appeared in the LS, MM, and SUB at 7 weeks old (Figure 3A), indicating that axonopathy happened first in these brain areas.

To further confirm axon vulnerability in the three brain areas, we quantitatively analyzed the spatiotemporal patterns of axonopathy in the LS, MM and SUB, respectively. In the three brain regions, the average distribution density of both the small and large axonopathic varicosities was significantly lower at 7 weeks old than that at 8 weeks old (Supplementary Figure S2). These results indicated that, before 8 weeks old, many new varicosities started to emerge and grew to a larger size in the LS, MM, and SUB during aging of the AD mouse model. Interestingly, the distribution density of small axonopathic varicosities continued to increase after 8 weeks old, while the large ones showed no difference at 16 weeks old than that at 8 weeks old in both the LS and SUB (Supplementary Figure S2). These results showed that although many new cases of small axonopathic varicosities emerged after 8 weeks old, the large ones stopped growing, indicating that large axonopathy may be saturated or disappear in the LS and SUB during this period. Surprisingly, both the small and large swellings at 16 weeks old were significantly lower than those at 8 weeks old in the MM, especially the large ones declined more than 80% (Supplementary Figure S2). These results indicated that in the MM, after 8 weeks old, the disappearance rate of axonopathy was higher than its emergence rate.

Next, we compared the difference among the LS, MM and SUB. During 7 to 8 weeks old, the distribution density of both the small and large swellings was significantly higher in the MM than in both the LS and the SUB (Figure 3B), indicating that axons in the MM were more severely damaged than those in the LS and SUB. In addition, at 7 weeks old, the small varicosities in the LS was significantly lower than that in the SUB (Figure 3B). However, the LS contained more varicosities than the SUB at 8 weeks old (Figure 3B), indicating that the growth rate in the LS was higher than that in the SUB. On the other hand, the distribution density of axonopathic varicosities, at 16 weeks old, in the LS was significantly higher than that in the MM and the SUB (Figure 3B), indicating that axonopathy continued to rapidly emerge in the LS from 8 to 16 weeks old.

Overall, these results showed that the GFP-positive distal axons that projected to the LS, MM, and SUB were first affected and worsened during aging, especially axons projected to the MM were most severely damaged.

### The Relationship Between Axonopathy and Amyloid-β Plaques in the LS, SUB, and MM

**FIGURE 4:**
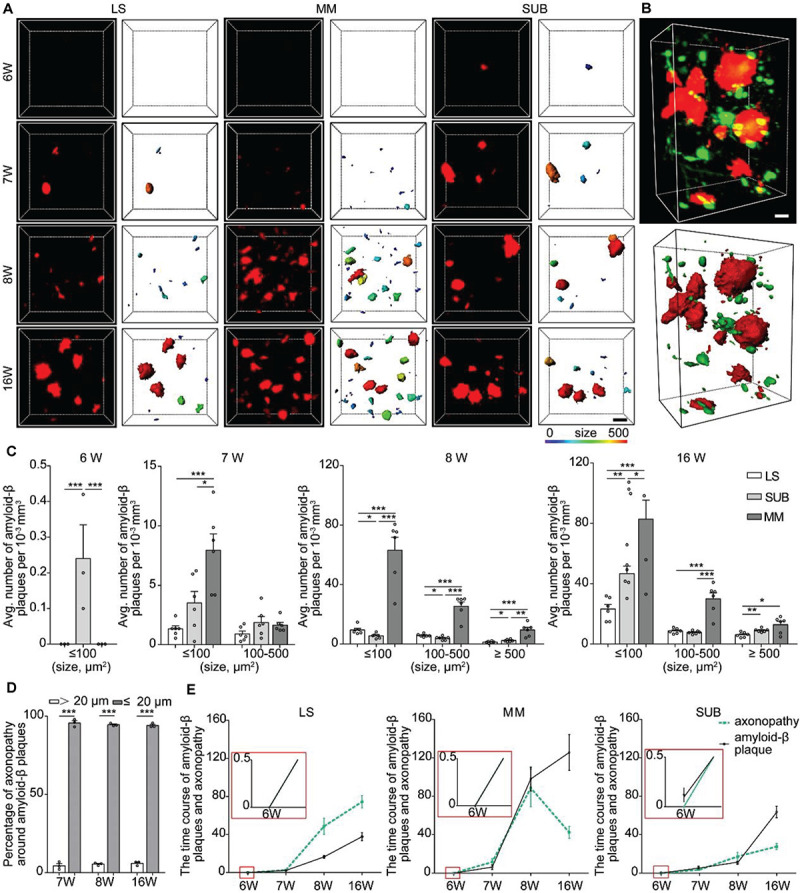
Spatiotemporal patterns of amyloid-β plaques and their relationship with axonopathy. **(A)** A 3D image of amyloid-β plaques in the LS, MM, and SUB of 5xFAD mice (left) at different ages. The surface render of amyloid-β plaques showed 3D morphology of plaques heterogeneity (right). Heat-map plots showed the size of amyloid-β plaques. Red = bigger size, blue = smaller size. Scale bars: 10 μm. **(B)** A 3D image of axonopathy around the Amyloid-β plaques (top). The surface render of amyloid-β plaques and axonopathy showed their 3D morphology (bottom). Red = Amyloid-β plaque, green = axonopathy. Scale bars: 10 μm. **(C)** Density comparison of amyloid-β plaques among the LS, MM, and SUB of 5xFAD mice at 6, 7, 8, and 16 weeks old (6W, *n* = 3; 7W, *n* = 6; 8W, *n* = 6; 16W, *n* = 6). **(D)** GFP-positive axonopathic varicosities occurred within or in the vicinity amyloid-β plaques (*n* = 3). **(E)** The time course between axonopathy and amyloid-β plaques during the early stage of the 5xFAD model. All error bars represent mean ± SEM and the significant differences were indicated by *p* value (Student’s *t*-test, *P < 0.05; **P < 0.01; ***P < 0.001).

Numerous studies have shown that amyloid-β plaque disrupts the transport of vesicles, neurotransmitters, and other cargos to cause axonopathy (Stokin et al., 2005; Gowrishankar et al., 2015). To determine whether amyloid-β plaque contributes to axon vulnerability in the LS, SUB and MM, we analyzed the spatial distribution of the plaques in the LS, MM, and SUB before and after axonopathy formation. We observed that a small number of amyloid-β plaques only appeared in the SUB, but not in other brain regions at 6 weeks old (Figure 4A), indicating that amyloid-β plaques formed earlier than axonopathy and may be a cause for it. At 7 weeks old, a small amount of amyloid-β plaques also appeared in the LS and MM (Figure 4A). Quantitative analysis showed that, from 7 to 16 weeks old, the density of amyloid-β plaques in the LS and SUB progressively increased (Figure 4A and Supplementary Figure S3). More importantly, in the MM (Figure 4A and Supplementary Figure S3), the density of amyloid-β plaques at 8 weeks old was significantly higher than that at 7 weeks old, while there was no difference between 8 and 16 weeks old. These results indicated that the accumulation of amyloid-β plaque further increased in the LS and SUB, while saturation may have been reached before 16 weeks old in the MM.

Next, we quantified the difference of amyloid-β plaques among the LS, MM and SUB. The results showed that, at 7 weeks old, the density of the small amyloid-β plaque was significantly higher in the MM than in the LS (Figure 4C), indicating that amyloid-β plaques formed faster in the MM than in the LS. After 8 weeks old, the density of amyloid-β plaques in the MM was significantly higher than that in the LS and SUB (Figure 4C). These results showed that spatial distribution of amyloid-β plaques varied dramatically in different brain regions during the early stage of 5xFAD mice. More importantly, we observed that amyloid-β plaques caused more serious damage to the MM than to the LS and SUB. To further explain the effect of amyloid-β plaque on axonopathy, we examined the nature and degree of axonopathy within and near amyloid-β plaques. We found that the axons developed swellings or spheroids in close proximity to amyloid-β plaques (Figure 4B). These pathological changes were consistent with previously described dystrophic neurites (Tsai et al., 2004; Blazquez-Llorca et al., 2017). Regardless of brain regions or size of amyloid-β plaques, most cases of axonopathy (95.97 ± 0.013 % at 7 weeks old, 94.52 ± 0.010 % at 8 weeks old and 94.04 ± 0.010 % at 16 weeks old) occurred in close proximity to amyloid-β plaques (within 20 μm) in the 5xFAD/GFP mice (Figure 4D). Furthermore, the time course between axonopathy and amyloid-β plaques showed that, after amyloid-β plaques formed, axonopathy increased significantly accompanied by the accumulation of amyloid-β plaques in the LS and SUB (Figure 4E). However, axonopathy dropped significantly when amyloid-β plaques reached a certain level in the MM (Figure 4E). Thus, our data indicated that amyloid-β plaque can lead to or contribute to axonopathy within or in the vicinity of it during the early stage of an AD model.

### MM Axons of SUB Projection Neurons Are Susceptible to Pathology

**FIGURE 5:**
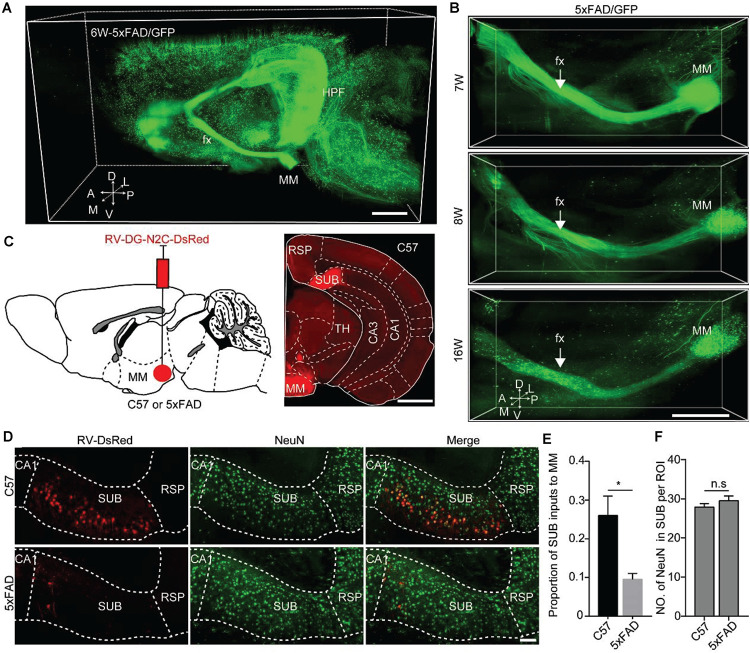
Quantitative analysis revealed that MM received monosynaptic input from SUB in 5xFAD and WT. **(A)** A 3D-reconstructed image of GFP-positive neurons and fibers in the hemisphere brain from a 6-week-old 5xFAD/GFP mice. A, anterior; D, dorsal; L, lateral; M, medial; P, posterior; V, ventral. Scale bars: 1 mm. **(B)** A 3D-reconstructed image of axons along the fornix projected to MM in the 7-, 8-, and 16-week-old 5xFAD/GFP mice, respectively. White arrow indicated the fornix. A, anterior; D, dorsal; L, lateral; M, medial; P, posterior; V, ventral. Scale bars: 1 mm. **(C)** Schematic diagram illustrating injection of RV-DG-N2C-DsRed in the MM of a 15-week-old C57 mice or 5xFAD mice to label direct input neurons from the SUB projecting to the MM (left), and characterization of injection site in the MM (right). Scale bars: 1 mm. **(D)** MM received direct input from the SUB, and immunostaining against NeuN in the SUB in 16-week-old C57 mice and 5xFAD mice. Scale bars: 100 μm. **(E)** Quantitative analysis of the proportion of the SUB inputs to the MM between 16-week-old C57 mice and 5xFAD mice. **(F)** Comparison of NeuN-positive neurons in the SUB between 16-week-old C57 and 5xFAD mice (*n* = 4 for each group). All error bars represent mean ± SEM and the significant differences were indicated by *p* value (Student’s *t*-test, *P < 0.05; n.s., nonsignificant).

The axonopathy spatiotemporal map, as well as the observation of the relationship between axonopathy and amyloid-β plaques, showed that the axons of GFP-positive neurons that projected to the MM were damaged more severely than other brain regions during early stage of 5xFAD mice. The MM is one of the core brain regions connecting the hippocampal formation to the rest of the Papez memory circuit, which mainly originates from the SUB (Vann and Aggleton, 2004; Vann, 2010). Thus, we hypothesized that axonopathy in the MM might locate on the axons of projection neurons of the SUB during the early stage of the AD mouse model. To validate this hypothesis, we performed whole-brain three-dimensional reconstruction in the 6-week-old 5xFAD/GFP mice. We found that a large number of the MM axons originated from GFP-positive neurons in the hippocampal formation and aggregated along the ependymal border of the fimbria and extended along the fimbria-fornix system, a major input-output pathway of the hippocampal formation (Figure 5A). The vast majority of these fibers ran along the fornix efferent to the MM (Figure 5A), indicating that the MM received dense projections from the hippocampal formation, as reported in previous studies (Vann and Aggleton, 2004; Porrero et al., 2010; Leroy et al., 2018). In particular, continuous three-dimensional datasets showed that the axons bearing pathology in the MM formed lesions in the fornix after axonopathy developed in the MM (Figure 5B), indicating retrograde degeneration of axons in the MM, which was consistent with the previous study (Ballinger et al., 2016).

To further validate the input brain areas of the MM, we injected RV-DG-N2C-DsRed into the MM in 15-week-old control and 5xFAD mice, respectively (Figure 5C). After 7 days, we found that the number of RV-labeled projection neurons in the SUB of the control mice were significantly higher than that in the SUB of the 5xFAD animals (Figures 5D,E). These results indicated early degeneration of the MM axons originating from the SUB projection neurons in the AD mouse model. To confirm that the decrease of RV labeled neurons in the SUB in the AD mouse model is due to retrograde axon degeneration rather than neuronal loss, we used immunochemical staining against NeuN to quantify the neurons in the SUB. We found that the number of NeuN in the SUB was similar between control and 5xFAD mice (Figure 5F). These results indicated that in 16-week-old 5xFAD mice, there was no significant neuronal loss in the SUB, however, the axons from the SUB to the MM already started to degenerate.

### Novel-Object Recognition Memory and Object-Location Memory Were Imparied in 5xFAD Mice

**FIGURE 6:**
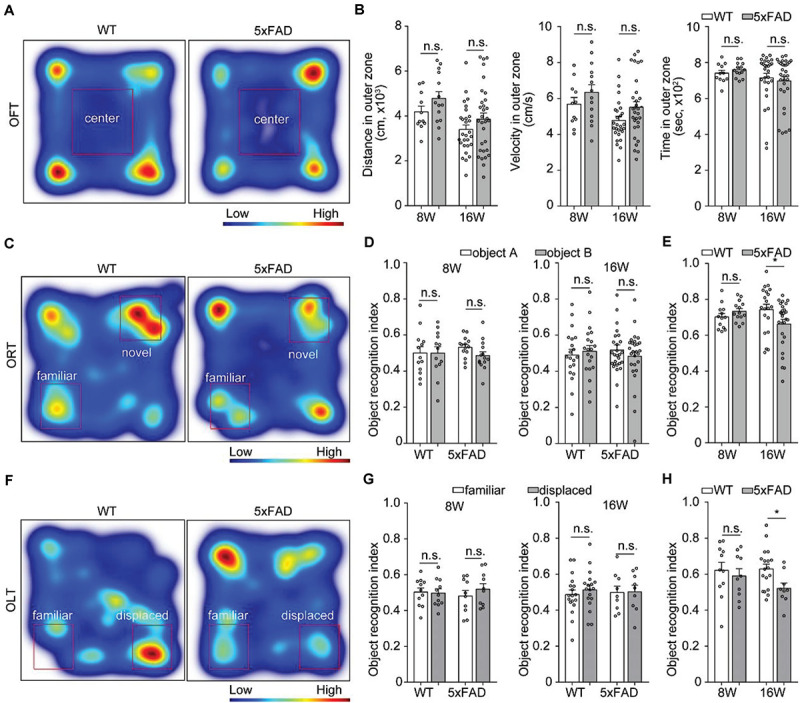
Behavioral performance in 5xFAD mice. **(A,C,F)** Heat-map plots showed the averaged cumulative time that 5xFAD and WT mice at 8 and 16 weeks of age spent in different parts in the test session of the OFT, ORT, and OLT, respectively. Red = more time, blue = less time. **(B)** There was no difference in the distance, time and velocity in the outer zone of an OFT between 5xFAD mice and their wild-type littermates mice (8W, *n* = 14 for 5xFAD, *n* = 11 for WT; 16W, *n* = 31 for 5xFAD, *n* = 28 for WT). **(D,G)** There was no statistical difference in time spent to explore one of the objects during the familiarization session in the ORT (8W, *n* = 14 for 5xFAD, *n* = 13 for WT;16W, *n* = 27 for 5xFAD, *n* = 20 for WT) and OLT (8 W, *n* = 10 for 5xFAD, *n* = 11 for WT; 16 W, *n* = 9 for 5xFAD, *n* = 18 for WT) were observed for 5xFAD compared to their wild-type littermates at 8 and 16 weeks of age, respectively. **(E,H)** During the test session, the 5xFAD mice explored significantly less the novel object and displaced object compared with the wild-type littermates at 16 weeks of age. However, there was no difference between the 5xFAD mice and the wild-type littermates at 8 weeks of age. All error bars represented mean ± SEM and the significant differences were indicated by *p* value (Student’s *t*-test, *P < 0.05; n.s., non-significant).

Previous studies showed spatial working memory by 6 months of age (Jawhar et al., 2012). To assess behavioral performance when the axons that projected to MM started to degenerate, we tested 8- and 16-week-old 5xFAD mice on OFT, ORT and OLT. In the OFT, the traveling distance, mean velocity and time in outer zone between wild type mice and 5xFAD mice were similar at the age of 8 and 16 weeks, respectively (Figures 6A,B), which indicated that the locomotion ability of 5xFAD mice did not alter compared to the wild type mice. However, compared with the wild type mice, 5xFAD mice at 16 weeks of age, but not at 8 weeks of age spent less time to explored both the novel object in the ORT (Figures 6C–E) and displaced object in the OLT (Figures 6F–H). These results suggested that 5xFAD mice at 16 weeks of age exhibited novel-object recognition memory and object-location memory dysfunction in the early stage.

## Discussion

In this study, to investigate the spatiotemporal variation of axonopathy in the 5xFAD mouse model, we obtained whole-brain datasets of GFP-positive neurons in the early stage of 5xFAD/GFP mice using the fMOST system. Axonopathy was identified as localized swellings on axons and first appeared in the LS, SUB, and MM at 7 weeks old, which was earlier than that reported in previous studies (Tsai et al., 2004; Adalbert et al., 2009; Blazquez-Llorca et al., 2017). These swellings appeared in many brain regions, including the isocortex, olfactory area, hippocampal formation, cortical subplate, striatum, thalamus, hypothalamus and fiber tracts. The progression of axonopathy continued to occur during aging in most brain areas. However, the number of swellings in the MM dropped significantly after 8 weeks old. Unlike the continuous accumulation of amyloid-β plaques, the progression speed of axonopathy from 7 to 8 weeks old was high, and declined from 8 to 16 weeks old. Taken together, we created a spatiotemporal axonopathy map of GFP-positive neurons, and demonstrated that the axonopathy was more likely to occur in specific neural pathways rather than appeared universally during the early stage of the AD mouse model.

Previous studies have showed that axons from the neocortical and hippocampal projection neurons were damaged in AD mouse models (Tsai et al., 2004; Blazquez-Llorca et al., 2017). However, in these studies, only a small number of brain areas were reported. It is challenging to study the temporal and spatial progression of axonopathy in three-dimensions. A study on AD reported the three-dimensional acquisition of senile plaques, neurofibrillary tangles, and axons using CLARITY in combination with light-sheet microscopy (Ando et al., 2014). However, the progression of axonopathy has yet to be reported. In the present study, we acquired the whole-brain datasets through fMOST with a resolution of 0.32 × 0.32 × 2 μm^3^ and systematically analyzed the spatiotemporal axonopathy patterns in the early stage of 5xFAD/GFP mice. Our results showed that axonopathy first appeared in the LS, SUB, and MM in 7-week-old 5xFAD/GFP mice, followed by other brain areas at later time points.

The LS, SUB, and MM are involved in the regulation of advanced functions, such as anxiety, object recognition, and memory (Vann and Aggleton, 2004; Calhoon and Tye, 2015; Matsumoto et al., 2018). We comprehensively analyzed the spatial distribution of axonopathy in the LS, SUB, and MM in 5xFAD/GFP mice younger than 16 weeks old. We confirmed that axonopathy can manifest as different sizes with swellings, ranging from 28 μm^2^ to more than 100 μm^2^. The axonopathy progression pattern is similar to that of amyloid-β plaque propagation (Canter et al., 2019).

Amyloid-β production and deposition have been reported to be involved in the regulation of axonal transport. In particular, Amyloid-β(42) was shown to damage the stability of tubulin and the normal function of motor proteins, which leads to cargo detachment from the motor proteins and causes axonopathy (De Vos et al., 2008; Sadleir et al., 2016). Amyloid-β plaques first appeared in the SUB, earlier than axonopathy, indicating that the plaques may be a cause of axonopathy. Furthermore, three-dimensional analysis confirmed that axonopathy occurred within or in the vicinity of amyloid-β plaques. Our results (Figure 4), as well as previous study (Canter et al., 2019) showed that in the 5xFAD mouse model, the accumulation of amyloid-β plaques in the MM was much more serious than in other brain areas. The high burden of amyloid-β plaques may cause serious damage to axons. Considering the decreased number of swellings in the MM from 8 to 16 weeks old, we reasoned that, when amyloid-β plaques reach its peak in one brain area, its axons quickly degenerate, causing neuronal loss in upstream regions. In fact, many proteins that cause hereditary AD, including amyloid-β precursor protein (Szpankowski et al., 2012), presenilin 1, presenilin 2 (Gunawardena et al., 2013) and β-secretase (Lee et al., 2005) can induce axonal transport defects and lead to the development of axonopathy. Thus, further studies are needed to identify the exact mechanisms of causing this condition.

Our three-dimensional reconstruction and retrograde RV tracing data showed that MM axons projecting from SUB projection neurons were severely damaged that just before the behavior disorder occurred. Previous studies have shown that the MM mainly receives inputs from the SUB, whose damage can lead to object recognition and spatial memory issues (Roy et al., 2017; Matsumoto et al., 2018). This pathway-specific impairment may be related to early symptoms of AD mouse model and AD patients such as spatial memory impairment.

It is noteworthy that the Thy1-GFP-M mouse line often shows Golgi staining-like labeling and does not provide strong cortical labeling (Feng et al., 2000). So, no all axonopathy could be labeled by GFP. This method may be flawed in that it can not distinguish all the lesions, nor can it describe the origin of the earliest axonopathy. However, in the present study, we found that axonopathy first occurred in the LS, SUB, and MM, which are the downstream brain areas of the hippocampus. Meanwhile, we found that the formation of axonopathy in these regions occurred very early (7 weeks old) that immediately after the formation of the amyloid-β plaques in the brain of 5xFAD (6 weeks old). Previous study also showed that the MM was the first brain area affected by AD development (Canter et al., 2019), which was consistent with our present study. Not only did we find that the LS, SUB, and MM were the first regions to exhibit axonopathy, we also identified the SUB-MM pathway was affected. Maintaining the normal structure and functions of the SUB-MM pathway may help in the treatment of early symptoms of AD. Finally, it is also noteworthy that all transgenic AD mouse models can be artificial in their genetic constitution. The progression of axonopathy was caused not only by the deposit of amyloid-β but also the overexpression of APP in 5XFAD mouse model. In the previous study, Canter et al proved that the temporal and spatial progression of amyloid-β in 5XFAD mouse brain is biologically relevant and region-specific rather than an artifact of the mouse transgene (Canter et al., 2019). Similarly, we found that the progression of axonopathy is highly related to the deposit of amyloid-β (Figure 4). Considering the artificial thy1 promoter used in 5XFAD mice is widely expressed across the brain (Canter et al., 2019), it is unlikely that the neural pathway specific progression of axonopathy is caused by the universal overexpression of APP. Nevertheless, it is unlikely that the rapid accumulation of amyloid-β in 5XFAD mouse brain can fully mimic peptide-mediated effects in the human brain, in which the disease evolves over years and decades and several pathologies develop in a time- and brain-region-dependent manner. Although we provided an axonopathy map and identified the neural pathway that was vulnerable to AD development in a mouse model, whether these knowledge can be applied to the treatment of patients and clinical research needs to be further investigated. At this stage, these data still provides invaluable insights into AD pathogenesis and therapeutic strategies.

## Conclusion

In this study, we created a spatiotemporal axonopathy map in early stage of 5xFAD/GFP mice and revealed that the axons of GFP-positive neurons showed an area-specific aggravation over time. Our results indicated that among GFP labeled axons, GFP labeled axonopathy underwent early alterations in the LS, SUB, and MM in the 5xFAD/GFP mouse model. We also found that the SUB-MM pathway was vulnerable to AD development in 5xFAD mouse model. Further investigation of the functions of the SUB-MM pathway and how its dysfunction leads to early symptoms of AD is needed. Our findings may help understand the development of AD and provide a novel target for the treatment of early symptoms of AD.

## Data Availability Statement

The raw data supporting the conclusions of this article will be made available by the authors, without undue reservation.

## Ethics Statement

The animal study was reviewed and approved by the Animal Care and Use Committee of Huazhong University of Science and Technology.

## Author Contributions

HG and XL conceived and designed the study. JZ, XL, and BL performed the experiments. ZD performed the whole-brain data acquisition. AL performed the imaging processing. JZ, XL, and HG wrote the manuscript. All authors contributed to the article and approved the submitted version.

## Conflict of Interest

The authors declare that the research was conducted in the absence of any commercial or financial relationships that could be construed as a potential conflict of interest.
