# Supplementary material for: Whole-Brain Three-Dimensional Profiling Reveals Brain Region Specific Axon Vulnerability in 5xFAD Mouse Model
Source: Front Neuroanat. 2020 Nov 26;14:608177. doi: 10.3389/fnana.2020.608177 (PMC7726261; doi:10.3389/fnana.2020.608177)
Supplement: Supplementary file 1 [file Data_Sheet_1.docx]

**Supplementary Materials**

**1 Supplementary Table: Abbreviation of brain regions**

| ACA | Anterior cingulate area | MM | Medial mammillary nucleus |
| --- | --- | --- | --- |
| ACB | Nucleus accumbens | MO | Somatomotor areas |
| act | anterior commissure, temporal limb | mPFC | medial prefrontal cortex |
| alv | alveus | NLOT | Nucleus of the lateral olfactory tract |
| AON | Anterior olfactory nucleus | OLF | Olfactory areas |
| BLA | Basolateral amygdalar nucleus | ORB | Orbital area­ |
| CA1 | Field CA1 | PA | Posterior amygdalar nucleus |
| CA3 | Field CA3 | PF | Parafascicular nucleus |
| cc | corpus callosum | PG | Pontine gray |
| CEXsp | Cortical subplate | PL | Prelimbic area |
| CL | Central lateral nucleus of the thalamus | RE | Nucleus of reuniens |
| CLA | Claustrum | RSP | Retrosplenial area |
| CP | Caudoputamen | RT | Reticular nucleus of the thalamus |
| DG | Dentate gyrus | SS | Somatosensory areas |
| EPv | Endopiriform nucleus, ventral part | SSp | Primary somatosensory area |
| fi | fimbria | st | stria terminalis |
| ­fx | columns of the fornix | STR | Striatum |
| HPF | Hippocampal formation | SUB | Subiculum |
| HY | Hypothalamus | SuM | Supramammillary nucleus |
| ILA | Infralimbic area | TH | Thalamus |
| Int | internal capsule | TR | Postpiriform transition area |
| LD | Lateral dorsal nucleus of thalamus | VAL | Ventral anterior-lateral complex of the thalamus |
| LGd | Dorsal part of the lateral geniculate complex | ZI | Zona incerta |
| LS | Lateral septal nucleus |  |  |

**2. Supplementary Figures**


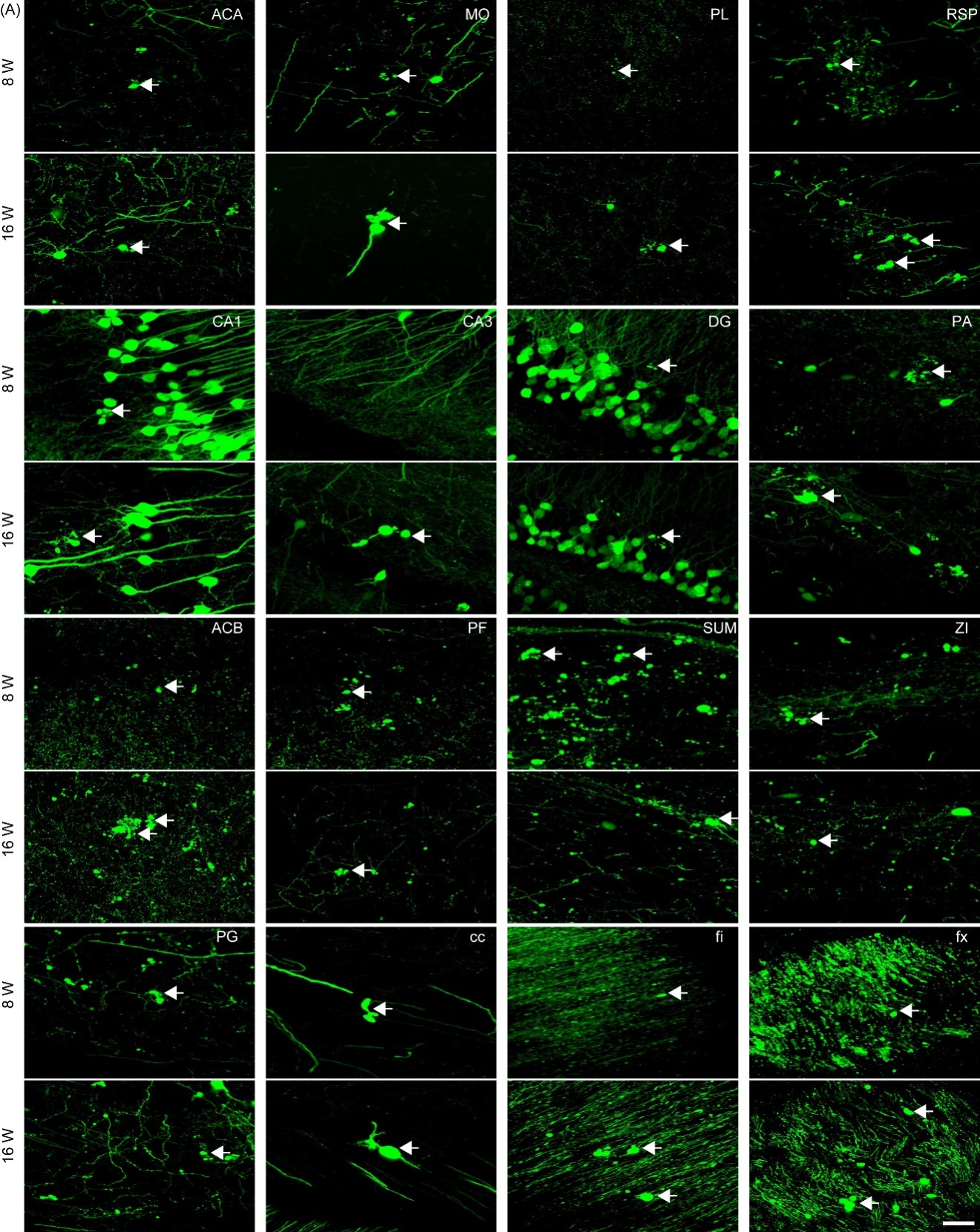


**Supplementary Figure1.The distribution of axonopathy in whole brain.**

(A)Maximum intensity protection of the axonopathy in representaive brain regions from 8 and 16-week-old 5xFAD/GFP mice. Representative axonopathy was highlighted with white arrow. The thickness of the projection was 20 µm. Scale bars: 20 µm.


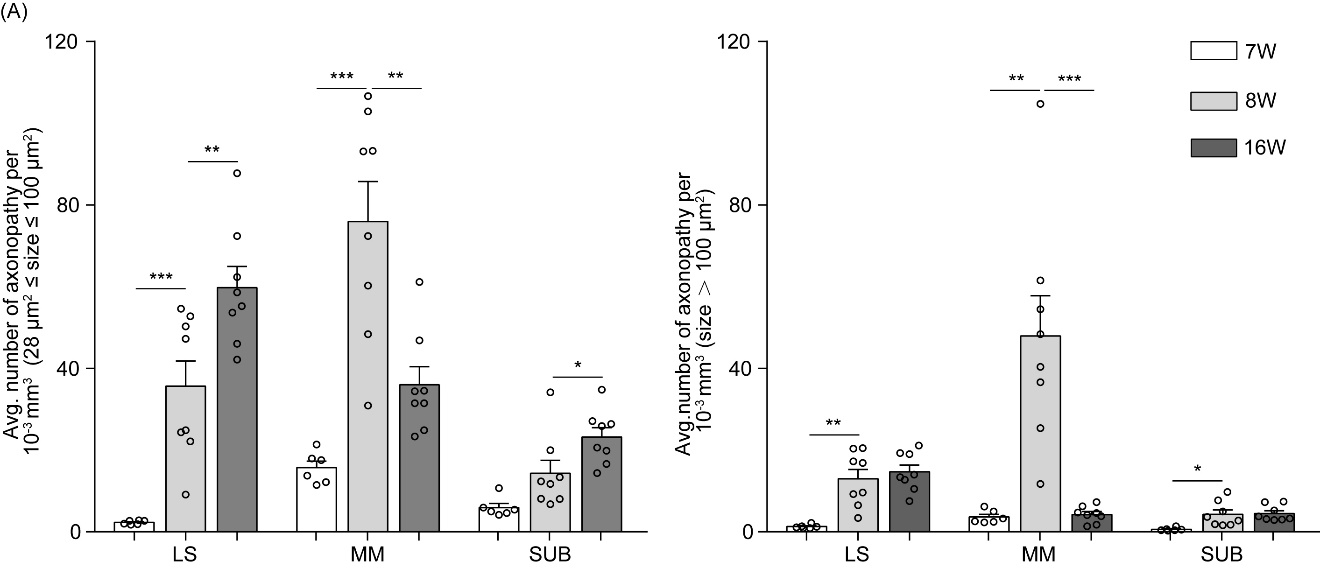


**Supplementary Figure. 2 The spatiotemporal patterns of axonopathy.**

(A)The average density of axonopathyin the LS, MM and SUB at 7, 8 and 16-week-old5xFAD/GFP mice, respectively (n = 3 for each group).All error bars represent mean ± SEM and the significant differences were indicated by p value(Student’s t-test, * P < 0.05; ** P < 0.01; *** P < 0.001).


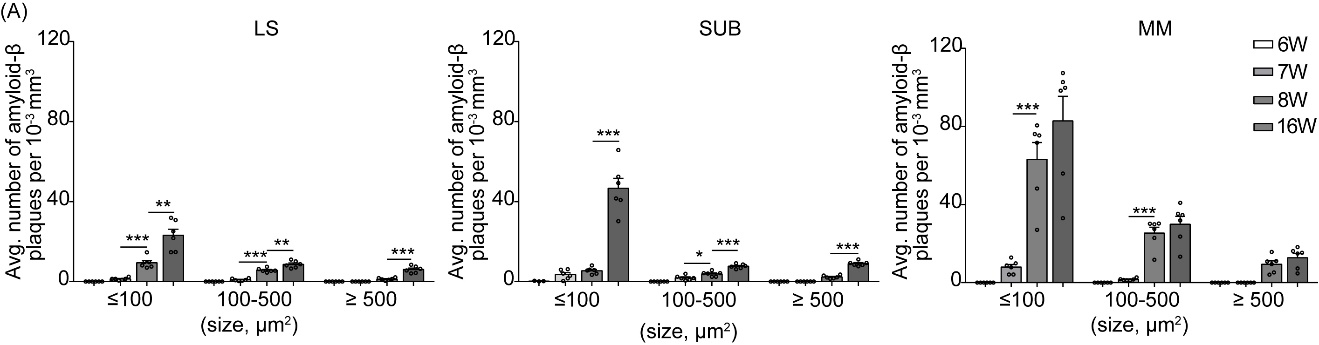


**Supplementary Figure. 3 The spatiotemporal patterns of amyloid-β plaques.**

(A)The average density of amyloid-β plaques in the LS, MM and SUBat 7, 8 and 16-week-old5xFAD mice,respectively (n = 3 for each group).All error bars represent mean ± SEM and the significant differences were indicated by p value (Student’s t-test, * P < 0.05; ** P < 0.01; *** P < 0.001).
